# Nutritional impact of nano zeolite, probiotic, and fatty acids as feed additives on health status of Nile tilapia (*Oreochromis niloticus*)

**DOI:** 10.1038/s41598-023-50034-2

**Published:** 2023-12-20

**Authors:** Manar Bahaa Abd Elshafy, Asmaa Ibrahim Mohamed Abd EL-Monem, Ibrahim M. Khattab, Sabreen E. Fadl, Galal Abou Khadiga

**Affiliations:** 1 Department of Animal and Fish Production, Faculty of Desert and Environmental Agriculture, Matrouh University, Matrouh, Egypt; 2 Department of Animal and Fish Production, Faculty of Agriculture, Alexandria University, Alexandria, Egypt; 3 Biochemistry Dept, Faculty of Veterinary Medicine, Matrouh University, Matrouh, Egypt; 4 Department of Poultry Production, Faculty of Desert and Environmental Agriculture, Matrouh University, Matrouh, Egypt

**Keywords:** Biochemistry, Physiology

## Abstract

For human consumption, fish is a good and affordable source of several crucial elements. Growing aquaculture management and output is always necessary. Therefore, this study was designed to evaluate the effect of probiotics, nano zeolite, and/or medium chain fatty acids (MCFA) on fish health and the chemical composition of the fish body. The experimental fish were distributed into eight groups. T1: Control group fed the basal diet without feed additives, T2: Nano zeolite at a rate of 2 mg/kg diet, T3: *Pedococcus* at a rate of 2 gm kg diet, T4: Medium chain fatty acids used according to produced company recommendation at a rate of 3.5 gm/kg diet, T5: Nano zeolite (2 mg/kg diet) + *Pedococcus* (2 mg/kg diet), T6: Nano zeolite (2 mg/kg diet) + Medium chain fatty acids (3.5 gm/kg diet), T7: *Pedococcus* (2 mg/kg diet) + Medium chain fatty acids (3.5 gm/kg diet), and T8: Nano zeolite (2 mg/kg diet) + *Pedococcus* (2 mg/kg diet) + Medium chain fatty acids (3.5 gm/kg diet). The obtained results showed an insignificant difference in the body composition of Nile tilapia fish fed feed additives alone or in combination. Moreover, the feed additives did not affect the health status of fish, as indicated by normal liver and kidney functions.

## Introduction

More than half of the world's population relies on fish as a source of dietary protein since it is one of the best and most affordable sources of lean meat^1,2^. Fish production and nutrition have received a lot of attention during the past 10 years^3,4^. The ability of farmed fish to demonstrate their genetic capacity for growth and reproduction is largely dependent on nutrition. Fish feed prices have risen, and there is a shortage, which has made it necessary to look for alternatives more actively. As a result, additives must be added to the fish meals^5^. Especially after the appearance of bacterial resistance to some antibiotics that are used either for treatment or as growth promoters^6–9^, the dispersal of contaminants that harm the ecosystem^9,10^ and the possible danger of antibiotic residues in animal-derived meals^7,9^. Moreover, there are rigorous regulations on the use of antibiotics and chemotherapeutics in the aqua feed sector^11^. Thus, the use of low-cost live food supplements as feed additives is widely accepted and embraced. The most suitable live feed additives are probiotics, which are useful microorganisms for the host's nutrition. The growth performance is improved and the mortality rate is decreased when probiotics are added to the feed. Probiotics have the ability to strengthen fish immune systems and increase their resistance to disease^12^. On the other hand, dietary medium-chain fatty acids and zeolite are frequently employed in aquafeeds to promote growth and metabolism^13–17^. Therefore, this study was conducted to evaluate the positive effects of *Pediococcus*, medium chain fatty acids, and/or nano zeolite on body composition and their side effects on the health status of Nile tilapia fish.

## Materials and methods

### Experimental design and diet

Nile tilapia fish was used to evaluate the effect of nano zeolite, Pediococcus (*Pediococcus acidilactici*), and/or medium chain fatty acids on body composition and health status. The fish used were mono sex males (n = 240 with average body weight 7 ± 1 g/fish) and obtained from a local farm. After the acclimatization period, the fish were distributed into eight groups according to the feed additives. The experimental diet was prepared by well-mixing of the feeding ingredients (Table 1). Then, pellets were made with fish oil and water to create a dough that was 1–2 mm in size by using a laboratory pelletizer. Following preparation, the diets were dried at room temperature and stored at 4 ^O^C. In order to achieve homogenous mixing of the total amounts of the various diets with feed additives, tiny amount of the basal diet was mixed with the respective amount of the required feed additives at first in a small batch, followed by larger amount of the basal diet. The experimental groups were T1: Control group fed the basal diet without feed additives, T2: Nano zeolite at a rate of 2 mg/kg diet (Bashar et al.^18^**,** obtained from Nanotech company, Egypt for Photo-Electronics, City of 6th October, Al Giza Governorate, Egypt.), T3: *Pedococcus* at a rate of 2 gm kg diet (Fadl et al.^19^**,** obtained from Egavet company, Giza, Egypt as Bactocell®), T4: Medium chain fatty acids used according to produced company recommendation at a rate of 3.5 gm/kg diet (obtained from Egavet company, Giza, Egypt.), T5: Nano zeolite (2 mg/kg diet) + *Pedococcus* (2 mg/kg diet), T6: Nano zeolite (2 mg/kg diet) + Medium chain fatty acids (3.5 gm/kg diet), T7: *Pedococcus* (2 mg/kg diet) + Medium chain fatty acids (3.5 gm/kg diet), and T8: Nano zeolite (2 mg/kg diet) + *Pedococcus* (2 mg/kg diet) + Medium chain fatty acids (3.5 gm/kg diet). The experimental period lasted for 86 days, during which fish were fed twice daily. The aquarium water was partially changed with dechlorinated fresh water every day with oxygen, salinity, and pH at 5.8–6.1 ppm, 1.1–2‰, 7.4–8.1, respectively, and a temperature of around 24 °C.

**Table 1:** Ingredients and proximate composition (g/kg dry matter) of the basal diet.

Ingredient	Diets							
	T1	T2	T3	T4	T5	T6	T7	T8
Fish meal	250	250	250	250	250	250	250	250
soybean meal	300	300	300	300	300	300	300	300
Rice bran	100	100	100	100	100	100	100	100
Corn meal	100	100	100	100	100	100	100	100
Corn starch	130	129.998	128	126.5	127.998	126.498	124.5	124.498
Corn oil	50	50	50	50	50	50	50	50
wood charcool	30	30	30	30	30	30	30	30
Mineral permix^a^	10	10	10	10	10	10	10	10
Vitamin permix^b^	8.75	8.75	8.75	8.75	8.75	8.75	8.75	8.75
Ascorbic acid	1.25	1.25	1.25	1.25	1.25	1.25	1.25	1.25
Sodium alginate	20	20	20	20	20	20	20	20
Nano	–	0.002	–	–	0.002	0.002	–	0.002
Probiotic	–	–	2	–	2	–	2	2
Fatty acids	–	–	–	3.5	–	3.5	3.5	3.5
Proximate composition								
Moisture	80	78.5	81.8	82.5	76.3	83.6	75.6	84
Crude protein	312	312	313	311	314	312	312	315
Crude lipid	111	112	112	114	113	110	114	112

^a^ Mineral premix (mg/kg diet): Mg: 12.75; Ca: 72.85; Zn: 0.55; Mn: 0.25; Cal: 0.02; K: 5.00; Fe: 2.50; Cu: 0.08; CO: 0.05; Cr: 0.01; Na CI: 6.00.

^b^ The premix provided following amounts per Kg of feed: A: 960,000 lU; D_3:_ 160,000 lU; E: 0.8 g; K: 0.16 g; B_1_: 80 mg; B_2_: 0.32 g; B_6_: 0.12 g; pantothenic acid: 0.8 g; B_12_: 0.8 mg; Niacin: 1.6 g; Folic acid: 80 mg; Biotin: 4 mg; Choline chloride: 40 g.

T1: Control, T2: Nano zeolite, T3: Pedococcus, T4: Medium chain fatty, T5: Nano zeolite + Pedococcus, T6: Nano zeolite + Medium chain fatty acids, T7: Pedococcus + Medium chain fatty acids, and T8: Nano zeolite + Pedococcus + Medium chain fatty acids.

At the end, the blood samples were collected from each group to separate serum. The serum samples were used to evaluate the health status of the experimental fish. This evaluation was done by measuring liver and kidney function in addition to liver histopathology. Using Bio-Diagnostic Company kits, the serum samples (separated at 3000 rpm for 15 min) were used for measuring total protein, albumin, urea, and creatinine concentrations and aspartate aminotransferase (AST) and alanine aminotransferase (ALT) activities. Moreover, five fish were collected from each replicate/group to make a chemical analysis of the fish body. The chemical composition was determined according to AOAC^20^. All experimental procedures were carried out according to the National Institutes of Health (NIH) general guidelines for the care and use of laboratory animals and recommended by the Ethics of Animal Use in Research Committee (IACUC), Faculty of Agriculture, Alexandria University, Egypt (Approval No. MDPHD 0,201,707).

### Protein protective value (PPV%)

This parameter was calculated using the method of Nose^21^.

PPV can be calculated as follows:

$${\text{PPV}}=\frac{{\text{B}}-{\text{B}}0}{\mathrm{I }}\times 100$$

where B: Total body protein in the tested fish at the end of the experiment, ${\text{B}}0$: Total body protein at the start of experiment, I: Protein Intake of the test diet during the experiment.

### Hepatosomatic index (HSI)

Liver was dissected out of five fish per aquarium were used, weighted individually. Hepatosomatic index (calculated as g 100/g body weight) can be expressed as follows: -

$${\text{HSI}}=\frac{\mathrm{Liver weight}({\text{g}}) }{\mathrm{Body Weight}({\text{g}}) }\times 100$$

### Yield (%)

Fish which were used previously in HSI were used also for determination for yield can be calculated as follows:

$${\text{Yield}}\% = { 1}00 \, \times ({\text{Weight without gut}}/{\text{Final weight}})$$

### Histopathology

At 86 days, liver from five freshly killed fish was obtained from each replication (15 fish/group). Using standard techniques, tiny sections of spleen, liver, and intestine were dehydrated and imbedded in paraffin wax. After being sectioned at a thickness of 3 μm, they were stained with haematoxylin and eosin (HE) and seen under a light microscope^22^.

### Statistical analysis

The data were analyzed by one-way ANOVA using SPSS version 20. The data were expressed as mean ± SE significant statistically (*P* ≤ 0.05).

### Ethical Approval

All methods were carried out in accordance with relevant guidelines and regulations. The authors confirm that the study was carried out in compliance with the ARRIVE guidelines.

## Results

### Body composition

The impact of nano zeolite, fatty acids, and/or *pediococcus* inclusion in the diet of *O. niloticus* on their body composition has been shown in Table 2. No significant differences were recorded in the total moisture, crude protein, crude lipids, or ash of Nile tilapia fed the experimental diets.

**Table 2:** Body proximate composition of Nile tilapia of fish fed the experimental diets for 86 days “% on wet weight basis”.

Treatment	Moisture	Crude protein	Ether Extract	Ash
T1	74.0 ± 0.46	16.8 ± 0.30	4.74 ± 0.17	4.42 ± 0.14
T2	72.5 ± 0.96	17.9 ± 0.43	4.86 ± 0.30	4.69 ± 0.31
T3	74.4 ± 1.71	16.6 ± 1.13	4.37 ± 0.30	4.67 ± 0.27
T4	73.2 ± 0.59	17.4 ± 0.48	4.66 ± 0.20	4.75 ± 0.09
T5	72.8 ± 1.49	17.7 ± 0.84	4.71 ± 0.50	4.77 ± 0.17
T6	72.9 ± 0.80	17.7 ± 0.38	4.92 ± 0.22	4.51 ± 0.21
T7	73.5 ± 0.32	17.2 ± 0.09	4.51 ± 0.08	4.79 ± 0.14
T8	73.4 ± 0.17	17.3 ± 0.10	4.86 ± 0.14	4.51 ± 0.06

Values are means ± standard error.

### Protein productive value and survival rate

The impact of nano zeolite, MCFA, and/or *Pediococcus* inclusion in the diet of *O. niloticus* on their protein productive value and survival rate has been shown in Table 3. The results of the protein productive value in the T5 group were extensively increased against the control and probiotic (T3) groups*.* On the other hand, there was an unsignificant (*P* ≤ 0.05) difference between the nano (T2), MCFA (T4), T7, and T8 groups. The survival rate did not differ significantly (*P* ≤ 0.05) between groups.

**Table 3:** protein productive value and Survival Rate of Nile tilapia (*Oreochromis niloticus)* fed diets for 86 days.

Treatment	PPV	Survival rate
T1	24.6 ± 1.26^b^	96.7 ± 3.33
T2	29.1 ± 0.92^ab^	96.7 ± 3.33
T3	24.8 ± 2.17^b^	90.0 ± 0.00
T4	26.0 ± 1.01^ab^	90.0 ± 0.00
T5	30.7 ± 3.19^a^	90.0 ± 0.00
T6	28.7 ± 1.03^ab^	90.0 ± 5.77
T7	25.6 ± 0.41^ab^	90.0 ± 10.0
T8	28.3 ± 1.53^ab^	93.3 ± 6.67

Values are means ± standard error. Mean values with different letters at the same column significantly differ at (*P* ≤ 0.05).

### Yield and hepatosomatic index

Significant interactive effects of nano zeolite, MCFA, and/or *Pediococcus* inclusion in the fish diet were noticed in yield and hepatosomatic index as shown in Table 4 at the end of the experiment. The yield showed no significant (*P* ≤ 0.05) difference between the different groups. There was an improvement in the hepatosomatic index of the T7 group against the T6 group. Meanwhile, the results of the control, T5, and T8 groups showed improvement of the hepatosomatic index against the nano (T2) and probiotic (T3) groups. Moreover, there was an unsignificant (*P* ≤ 0.05) difference between the MCFA (T4) and T6 groups.

**Table 4:** Yield and Hepatosomatic index of Nile tilapia (*Oreochromis niloticus)* fed experimental diets for 86 days.

Treatment	Yield	HIS
T1	91.5 ± 0.28	2.67 ± 0.36^ab^
T2	92.1 ± 0.48	1.34 ± 0.22^c^
T3	92.6 ± 0.89	1.06 ± 0.23^c^
T4	91.8 ± 0.079	1.91 ± 0.19^bc^
T5	91.5 ± 0.47	2.27 ± 0.27^ab^
T6	91.6 ± 1.35	1.80 ± 0.34^bc^
T7	90.2 ± 1.29	2.84 ± 0.13^a^
T8	89.9 ± 1.58	2.67 ± 0.44^ab^

Values are means ± standard error. Mean values with different letters at the same column significantly differ at (*P* ≤ 0.05).

### Liver and kidney function

The impact of nano zeolite, MCFA, and/or *pediococcus* inclusion in the feed of fish on the serum biochemicals at 86 days has been shown in Table 5. The results of the AST (U/L) in the T8 group were extensively increased against the control, T2, T3, and T4 groups. On the other hand, there was an unsignificant (*P* ≤ 0.05) difference between treatments when used singly and the control group. Moreover, there were unsignificant differences between T5, T6, and T7 against each other. The results of ALT (U/L) in the T8 group were extensively increased against the control, T3, T4, and T7 groups. Meanwhile, the total protein (g/dl) and albumin showed that T8 group significantly (*P* ≤ 0.05) improved against the other groups. However, the results of the total protein and albumin were significantly improved in the T3, T6, and T7 groups against the control group. Meanwhile, the globulin significantly increased in the control and T2 groups against the other groups. The results of creatinine mg/dL and urea were extensively increased in the T8 group against T4 and T7 groups.

**Table 5:** Serum biochemical parameters of Nile tilapia fed experimental diets for 86 days.

Treatment	AST (U/L)	ALT (U/L)	Total protein (g/dl)	Albumin (g/dl)	Globulin (g/dl)	Creatinine mg/dL	Urea mg/dL
T1	14.0 ± 0.55^c^	33.7 ± 2.3^b^	3.55 ± 0.04^c^	2.10 ± 0.04^e^	1.45 ± 0.01^a^	0.37 ± 0.05^abc^	5.81 ± 0.37^d^
T2	16.0 ± 0.58^bc^	38.3 ± 1.20^ab^	3.67 ± 0.04^bc^	2.24 ± 0.05^de^	1.42 ± 0.01^a^	0.40 ± 0.02^abc^	7.11 ± 0.32^abc^
T3	14.0 ± 1.15^c^	34.3 ± 0.88^b^	3.74 ± 0.05^b^	2.40 ± 0.03^ cd^	1.34 ± 0.01^b^	0.37 ± 0.01^abc^	6.54 ± 0.09^ cd^
T4	13.7 ± 0.88^c^	33.7 ± 1.86^b^	3.70 ± 0.07^bc^	2.39 ± 0.13^ cd^	1.31 ± 0.06^b^	0.34 ± 0.03^bc^	6.69 ± 0.25^bcd^
T5	18.3 ± 0.88^ab^	37.0 ± 1.15^ab^	3.72 ± 0.06^bc^	2.52 ± 0.05^c^	1.20 ± 0.04^c^	0.42 ± 0.02^ab^	7.77 ± 0.45^ab^
T6	18.7 ± 1.20^ab^	37.3 ± 0.67^ab^	3.79 ± 0.08^b^	2.73 ± 0.09^b^	1.06 ± 0.01^d^	0.39 ± 0.03^abc^	6.96 ± 0.38^abc^
T7	17.0 ± 0.58^ab^	34.0 ± 0.58^b^	3.84 ± 0.05^b^	2.78 ± 0.06^b^	1.07 ± 0.01^d^	0.33 ± 0.01^c^	6.37 ± 0.28^ cd^
T8	19.0 ± 0.58^a^	49.3 ± 11.4^a^	4.10 ± 0.07^a^	3.07 ± 0.04^a^	1.03 ± 0.03^d^	0.45 ± 0.04^a^	7.88 ± 0.41^a^

Values are means ± standard error. Mean values with different letters at the same column significantly differ at (*P* ≤ 0.05).

### Histopathological findings

**Figure 1:**
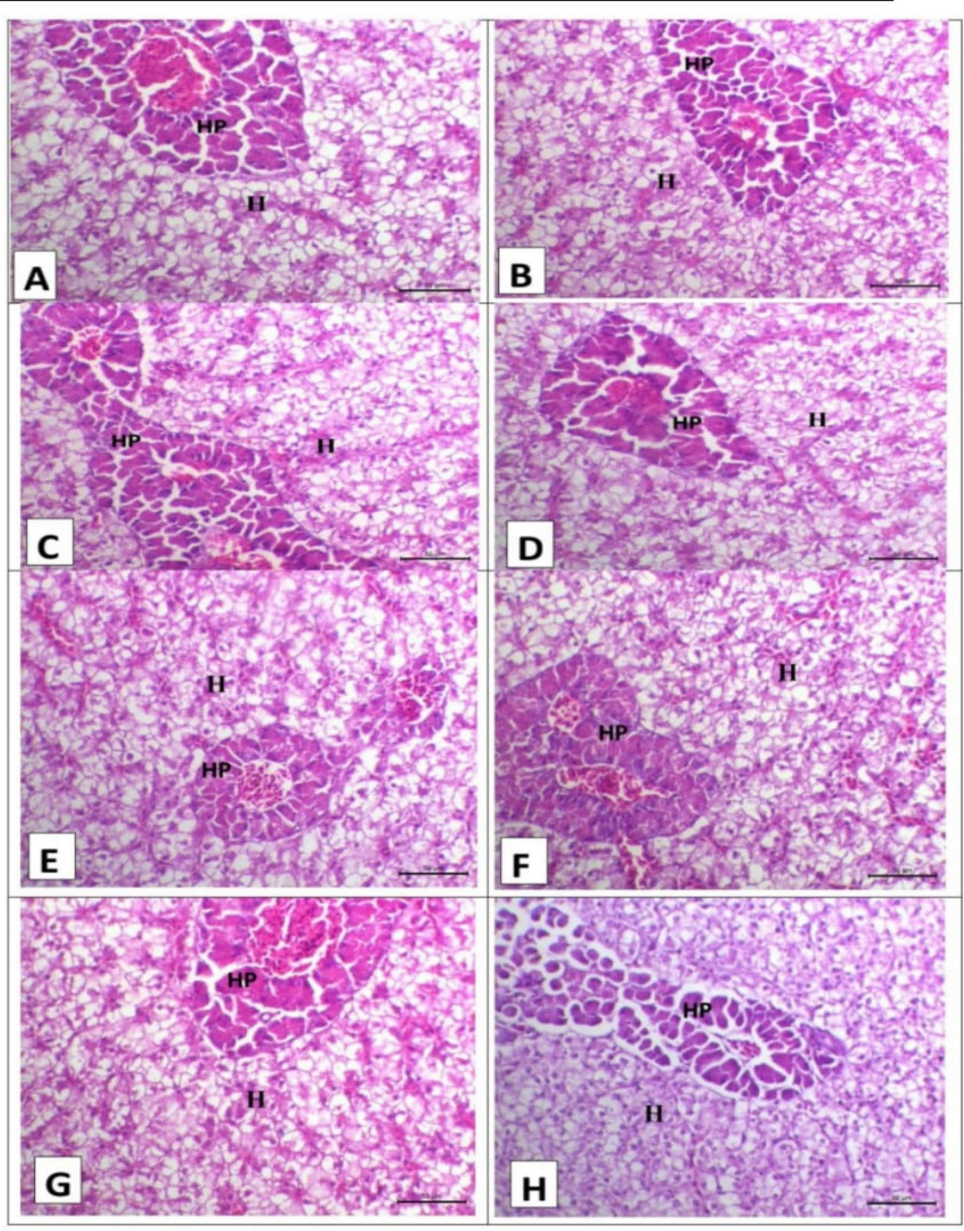
Representative photomicrograph of the hepatopancreas in different groups after 86 days of the experiment stained with H&E (X200, Scale bar = 50µ), where (**A**), (**B**), (**C**), (**D**), (**E**), (**F**), (**G**), and (**H**) are for the control group, T2 group, T3 group, T4 group, T5 group, T6 group, T7 group, and T8 group, respectively. All figures show normal hepatopancreatic histo-architecture where hepatocytes cords (H) and pancreatic acini surrounding central veins (HP).

The histopathological examination of the hepatopancreas of the Nile tilapia is illustrated in Fig. 1. The hepatopancreatic tissue of the control, T2, T3, T4, T5, T6, T7, and T8 groups showed normal hepato-pancreas and hepatic tissue (Fig. 1).

## Discussion

The shift of aquaculture operations towards intensification has put enormous pressure on the fishing industry. Therefore, new additives are being sought to improve growth in fish without affecting their health^23^. The results of dry matter, crude protein, and ash are consistent with the results of Eissa et al.^24^, who also reported that there wasn’t a significant difference in dry matter, crude protein, or ash among the various feed supplements. Nssar^25^ reported that no appreciable variations in the carcass composition of fish-fed diets containing zeolite were discovered. Similarly, Ullah et al.^26^ reported that the chemical compositions of the dorsal muscles and the entire body did not differ significantly across the groups fed a diet supplemented with lauric acid. Choi et al.^27^ reported that there is no effect of four functional feed additives on body composition in juvenile olive Flounder.‏ However, these results are incompatible with those of Attalla et al.^28^, who reported that the body composition of Nile tilapia fish fed feed additives showed a drop in fat percentage and an increase in protein content. Moreover, in contrast to Eissa et al.^24^, the body composition study showed no alterations in lipid contents with probiotic treatment. Similar findings were made by El-katcha et al.^29^ and Yones et al.^30^. These differences may be attributed to differences in fish species, types of feed additives, dose, source, and period of exposure. The results of the protein productive value, yield, and hepatosomatic index improved with the probiotic supplementation. This finding is confirmed by Patel et al.^31^. Abdelaty et al.^32^ reported the positive effect of the probiotic on the output of the Nile tilapia. A similar finding was reported by Shija et al.^33^. Moreover, Naiel et al.^34^ said that water probiotics in aquaculture improved productivity. However, Ali et al.^35^ and Ullah et al.^26^ noticed the positive effect of zeolite and medium-chain fatty acids alone on the protein productive value, yield, and hepatosomatic indx of Nile tilapia and black sea bream, respectively.

Plasma biochemistry measurements can be used to assess the health of fish^36,37^. Additionally, they serve as indicators for determining the health of fish after they had an additive-supplemented diet and have experienced challenges associated with fish husbandry^38^. The results of the serum ALT and AST when used as feed additives alone are compatible with the results of El-Kady et al.^39^, who reported the beneficial effect of probiotics as water additives on the serum biochemistry of Nile tilapia. Zeolite reversed the lead toxicity-induced rise in serum ALT and AST in Nile tilapia^40^. Moreover, Nile tilapia subjected to ammonia toxicity had their liver and kidney function enzyme levels restored by zeolite treatment^41^. Mansour et al.^42^ reported the beneficial role of zeolite on European sea bass hematobiochemical parameters. Fish-fed diets supplemented with 10 g/kg diet zeolite showed the lowest blood alanine and aspartate aminotransferase and alkaline phosphatase activity^25^.‏ Magouz et al.^43^ found that MCFA decreased the activity of AST and ALT in common carp.‏ Even when using the additives in combination with each other, there was no effect on serum biochemistry. This result is in harmony with the results of Simó-Mirabet et al.^44^, who reported that the combination of MCFA and probiotic did not alter the plasma protein of gilthead sea bream.‏ However, the combination of the three additives affected the activity of the AST and ALT. To the contrary, Choi et al.^27^ reported that there is no effect of four functional feed additives on serum biochemistry in juvenile olive Flounder.‏ This difference may be attributed to species differences. On the other hand, the humoral immune system relies heavily on serum proteins, albumin, and globulin, which also carry endogenous metabolites^45,46^. In this trial, the levels of serum proteins were increased with different feed additives. These findings are confirmed by the results of Kord et al.^47^, who reported that in fish treated with feed additives, levels of total blood proteins were significantly higher. The levels of blood proteins and albumin in Nile tilapia improved with the supplementation of some safe feed additives^48^. To the contrary, Choi et al.^27^ reported that combinations of four feed additives did not affect serum proteins. Regarding results of kidney function, serum creatinine and urea decreased when feed additives were used alone, as recorded by Youssef et al.^49^. As a result of the feed additive addition, serum total protein levels rose, while urea and creatinine levels fell, and ALT and AST activity decreased^50^. Similarly, Naser et al.^51^ reported the same results for creatinine and urea in Nile tilapia fish. However, the use of the combination of the three feed additives increased serum urea and creatinine. Magouz et al.^52^ found that ALT, AST, total protein, albumin, globulin, creatinine, uric acid, and urea were not significantly affected by the supplementation of a combination of feed additives. These differences may be attributed to the type of feed additive and dose.

The results of the serum liver function testes were confirmed by the results of liver histopathology, especially when used as a feed additive alone. Additionally, this study found a close association between liver microscopy findings and AST and ALT activity. These results are in agreement with Rašković et al.^53^. Hassaan et al.^54^ reported normal liver architecture and structure when using a nano zeolite-supplemented diet in Nile tilapia. The same findings were obtained by Zhang et al.^55^ when using medium-chain triglycerides in pigs. Moreover, the results of the combination of the three additives showed normal liver tissue, although the liver enzymes were elevated significantly against control. However, normally, minimal amounts of liver enzymes might be found in serum^56^. Moreover, these enzymes are released into the blood, where the higher amounts can be detected by any procedure that results in the loss of hepatocyte membrane integrity or necrosis^57^. But in this trial, no pathological lesion was detected in the liver tissue. These results may be attributed to an increase in the metabolism of liver tissue. This is indicated by the results of the serum proteins. The serum levels of these enzymes may be influenced by a number of physiological and risk factors, including age, sex, body mass index, pubertal age, high triglyceride levels, insulin resistance, and blood sugar levels^58,59^.

## Conclusion

For human consumption, fish is a good and affordable source of several crucial elements. Growing aquaculture management and output is always necessary. Research on novel feed additives, such as the addition of probiotics, nano zeolite, and/or medium-chain fatty acids to fish feeds, is urgently needed in order to lower feed costs, maximize digestibility, and prevent the residual effects of hormones and antibiotics on fish muscles, which in turn affect people who eat the fish without side effects on the fish health.

## Data Availability

The datasets used and/or analyzed during the current study available from the corresponding author on reasonable request.
